# Targeting TOPK sensitises tumour cells to radiation-induced damage by enhancing replication stress

**DOI:** 10.1038/s41418-020-00655-1

**Published:** 2020-11-09

**Authors:** Katharine J. Herbert, Rathi Puliyadi, Remko Prevo, Gonzalo Rodriguez-Berriguete, Anderson Ryan, Kristijan Ramadan, Geoff S. Higgins

**Affiliations:** MRC Oxford Institute for Radiation Oncology, University of Oxford, Old Road Campus Research Building, Roosevelt Drive, Oxford, OX3 7DQ UK

**Keywords:** Tumour biomarkers, Oncogenes, Preclinical research

## Abstract

T-LAK-originated protein kinase (TOPK) overexpression is a feature of multiple cancers, yet is absent from most phenotypically normal tissues. As such, TOPK expression profiling and the development of TOPK-targeting pharmaceutical agents have raised hopes for its future potential in the development of targeted therapeutics. Results presented in this paper confirm the value of TOPK as a potential target for the treatment of solid tumours, and demonstrate the efficacy of a TOPK inhibitor (OTS964) when used in combination with radiation treatment. Using H460 and Calu-6 lung cancer xenograft models, we show that pharmaceutical inhibition of TOPK potentiates the efficacy of fractionated irradiation. Furthermore, we provide in vitro evidence that TOPK plays a hitherto unknown role during S phase, showing that TOPK depletion increases fork stalling and collapse under conditions of replication stress and exogenous DNA damage. Transient knockdown of TOPK was shown to impair recovery from fork stalling and to increase the formation of replication-associated single-stranded DNA foci in H460 lung cancer cells. We also show that TOPK interacts directly with CHK1 and Cdc25c, two key players in the checkpoint signalling pathway activated after replication fork collapse. This study thus provides novel insights into the mechanism by which TOPK activity supports the survival of cancer cells, facilitating checkpoint signalling in response to replication stress and DNA damage.

## Introduction

Owing to the uncontrolled and deregulated cell growth in tumours, endogenous replication stress is a common feature of cancer cells [1]. Oncogene activation can lead to the deregulation of DNA replication through various mechanisms, including changes in origin licencing and firing, fork progression or cell-cycle timing [2, 3]. Exogenous sources of replication stress include DNA damage (by ionising or UV radiation, topoisomerase inhibitors and secondary structure inducers) or substrate depletion (exhaustion of nucleotide pools, histones and DNA binding proteins, and inhibition of replication machinery) [2]. Replication stress impairs DNA transcription and impairs cell-cycle checkpoint control and can ultimately cause genomic damage in the form of localised DNA lesions [2, 3]. Consequently, cancer cells develop selective resistance to oncogene-driven replication stress, which enables them to tolerate higher levels of exogenous DNA damage than untransformed cells [1]. As a consistent and specific feature of tumorigenesis, replication stress represents a potential target for selective cancer therapy [4, 5].

ATR-CHK1-Cdc25 signalling is the predominant regulatory mechanism activated as a consequence of DNA damage sustained during active cellular replication [6]. CHK1 activation triggers a multi-stage signalling cascade, which stabilises stalled replication forks, regulates origin firing and delays G2/M progression by Cdk1/2 activation until such time as the defect is resolved [7]. When activation of the intra-S and G2/M checkpoint is impaired by inhibition of ATR-CHK1-Cdc25 signalling, under-replicated and damaged DNA progresses from G2 into mitosis, producing mitotic abnormalities and dysregulated cytokinesis [8].

T-LAK-originated protein kinase (TOPK) is a recently characterised mitogen-activated protein kinase, which is upregulated in many subtypes of cancer. As overexpression is associated with tumorigenic tissues, TOPK is a potential biomarker and selective target for cancer treatment, with a number of inhibitory agents currently in preclinical development. Although its precise role in malignant transformation is currently unknown, the phenotype for TOPK suppression in cancer cells is cell cycle related, causing G2/M checkpoint failure leading to cytokinesis defects, multipolar nuclei and enhanced apoptotic cell death occurring 48–72 h following DNA damage [9–11]. We have previously shown that TOPK depletion increases radiosensitisation [10], however, a potential mechanism for TOPK’s role in the protection against exogenous and endogenous DNA damage is still unclear.

TOPK’s function is thought to be largely confined to mitosis. Here we show that TOPK also plays an important role in S-phase progression and that depleting or inhibiting TOPK weakens resistance to radiation-induced DNA damage by sensitising tumour cells to replication stress. Our investigations demonstrate that TOPK directly interacts with CHK1 and Cdc25C during cell-cycle progression, and that suppression of TOPK leaves cancer cells vulnerable to DNA damage during CHK1-mediated checkpoint activation. We show that this phenotype can be exploited to improve the outcome of radiotherapy treatments with TOPK inhibition demonstrating a clear radiosensitising effect in an in vivo tumour growth delay model.

## Materials and methods

### Cell culture

Tumour cell lines (H460, Calu-6) were cultured in RPMI 1640 medium supplemented with 10% foetal bovine serum (Sigma). Cultures were maintained at 37 °C and 5% CO_2_ and were routinely tested for mycoplasma using MycoAlert testing kit (Lonza). All cell lines were acquired from the American Type Culture Collection and those grown beyond 4 months after purchase were regularly authenticated by short tandem repeat profiling (DNA Diagnostics Centre).

### Reagents

Mouse monoclonal anti-PBK (TOPK) was purchased from Sigma (SAB5300406). Anti-RPA70 (ab79398), RPA32 (76420), CldU (ab6326), γH2AX (phospho-Ser139; ab18311), Cyclin A2 (ab32386), Cyclin E1 (ab133266) and PBK (phospho-Thr9; ab194953) antibodies were from Abcam. Antibodies targeting the phospho-HpTGEKP motif were from Millipore (ABE319). Anti-BrdU/IdU was from BD Biosciences (BD347580), and CHK1 (Total; #2360), CHK1 (phospho-Ser345; #2348), Cdc25C (Total; 4688) and Cdc25C (phospho-Ser216; 4901) antibodies were from Cell Signalling Technology. Alexa Fluor conjugated goat anti-mouse, anti-rat and anti-rabbit antibodies were from Invitrogen. Unless otherwise stated, all other reagents were from Sigma.

### In vivo experiments

H460 (0.5 × 10^6^) or Calu-6 (1 × 10^6^) lung cancer cells were inoculated subcutaneously with matrigel (BD Biosciences) in CD-1 nude female mice at age 55–70 days. Animals were randomly assigned to each treatment group using a random number generator (EXCEL), but experiments thereafter were not blinded. A sample size of six mice per group was used and was calculated to have 90% power (standard deviation (SD) 25%, alpha 0.05) to detect an 80% difference in tumour volume, based on G*Power 3.1 [12] and our previous experience. Once xenografts had reached 100 mm^3^, mice were treated with Vehicle (5% DMSO/40% PEG400) or 20 mg/kg OTS964 via intraperitoneal injection on days 0 and 2. For the radiation treatments, fractionated doses of 2 Gy/dose were delivered to tumours on days 0, 1 and 2 with a Gulmay-320 cabinet irradiator (Gulmay) at a dose rate of 2.0 Gy min^−1^, using lead shielding. The project licence covering the animal work (PPL30/3395) was approved by the Oxford University Animal Welfare and Ethical Review Body and granted by the UK Home Office Animals in Science Regulation Unit under the Animals (Scientific Procedures) Act 1986.

### Transfection

Ambion Silencer Select siRNAs (Life Technologies) were transfected into cell lines using RNAiMAX transfection reagent (Invitrogen) at a concentration of 20 nM according to the manufacturer’s instructions. Experimental protocols were initiated 48–72 h following transfection, and knockdown (KD) efficiency was confirmed at the time of irradiation treatment by immunoblotting. The siRNAs used for this study have been published previously and have demonstrated effective TOPK KD without producing off-target effects [10].

### Colony formation assays

Cells were plated as single cell suspensions and left to attach for 8 h prior to irradiation. Colonies were grown for 7–14 days, stained with crystal violet and counted using an automated GelCount plate scanner (Oxford Optronix). The plating efficiency (PE) [PE = Average Colony Number/Cells plated] and the surviving fraction (SF) at a given IR dose was calculated using [SF = PE^IR^ Dose/PE^0Gy^]. The sensitisation enhancement ratio at an SF of 0.10 (SER_10_) was determined by linear quadratic modelling of survival data. The significance of differences between curves was calculated by two-way ANOVA, with survival as the dependent variable and irradiation doses and treatment conditions as the two independent variables.

### Drug treatment and irradiation

OTS964 (OncoTherapy Science, Inc.) and Nocodazole (Sigma) were dissolved in DMSO and used at concentrations stated. Hydroxyurea (HU; Sigma) was dissolved in water and used at a concentration of 1 mM unless otherwise stated. Irradiation for in vitro experiments was delivered at a dose rate of 1.938 Gy × min^−1^ using a GSR D1 caesium-137 irradiator (Gamma Service).

### Co-immunoprecipitation

Proteins were extracted from precleared whole-cell lysates prepared in RIPA lysis buffer (Thermo Scientific) with protease inhibitors (Roche) and phosphatase inhibitors (Sigma) by incubation with 25 μL of Protein A/G beads (Santa Cruz) preloaded with 2 μg of antibody for 5–6 h at 4 °C. Immunoprecipitated proteins were released from the beads by boiling in LDS sample buffer, and analysed by western blotting.

### Immunoblotting

Cell lysates were prepared in RIPA lysis buffer (Thermo Scientific) with protease inhibitors (Roche) and phosphatase inhibitors (Sigma) and protein concentration was determined using the BCA assay (Thermo Scientific). Proteins were separated by SDS-PAGE and transferred to PVDF membranes, which were probed by overnight incubation at 4 °C in primary antibody solution. Targets were detected via HRP-conjugated secondary antibodies exposed to chemiluminescence reagent (Millipore).

### Fluorescence microscopy

Cells were grown on coverslips at subconfluent densities and treatments initiated during logarithmic growth phases. At the relevant time points, nonchromatin bound nuclear protein was removed by preincubation of coverslips with CSK extraction buffer (100 mM NaCl, 300 mM Sucrose, 3 mM MgCl_2_, 10 mM PIPES (pH 7.0), 1 mM EGTA, 0.5% Triton X100) for 10 min on ice, after which samples were fixed with 4% paraformaldehyde for 15 min. Coverslips were blocked before incubation with primary antibody for 3 h, followed by the corresponding fluorescent secondary antibodies for 1 h at room temperature. Nuclei were counterstained with 0.5 μg/ml 4′,6-diamidino-2-phenylindole (DAPI; Sigma), and coverslips were mounted using Vectashield (Vector Laboratories). Z stack images were randomly acquired under identical parameters with a Zeiss LSM710 fluorescence microscope (Zeiss) using a ×63 objective for γH2AX, bromodeoxyuridine (BrdU) and RPA foci assessment (>10 fields per sample). Nuclear BrdU, γH2AX and RPA foci number were analysed using ImageJ software.

### DNA fibre assays

Cultured cells were incubated for 30 min with media containing IdU (25 mmol/L) followed by PBS wash and incubation with media containing CldU (250 mmol/L) for 30 min. Cell suspensions were pipetted onto glass microscopy slides and lysed for 10 min. DNA fibres were stretched by raising slides to an angle of 45° after which the samples were fixed overnight in acetic acid/methanol solution. Fibres were washed in PBS and denatured in 2N HCl followed by immunostaining. IdU was detected using anti-BrdU (mouse) primary antibody (BD) and Alexa Fluor488 anti-mouse secondary antibody (Invitrogen). CIdU was detected using anti-BrdU (rat) primary antibody (Abcam) and Alexa Fluor647 anti-rat secondary (Invitrogen). Images (>20 per condition) were randomised and obfuscated using ImageJ software prior to measurement.

### Flow cytometry

Cells were incubated with 20 μM BrdU for 30 min and then left unirradiated or exposed to 4 Gy IR. Immediately afterwards, BrdU was removed and cells were returned to 37 °C and, at different time points, floating and attached cells were collected together and fixed with ice cold 70% ethanol. Samples were incubated with 0.1 mg/ml pepsin in 2N HCl at room temperature in the dark for 20 min. Then, samples were incubated with mouse anti-BrdU (BD Bioscience) in 1% BSA/PBS for 2 h and with an Alexa Fluor conjugated secondary anti-mouse antibody for 1 h in the dark. Finally, samples were incubated with 25 μg/ml propidium iodide (PI) and 200 μg/ml RNase in PBS for 20 min, and analysed using FACSCalibur cytometer (Becton Dickson). S-phase length was determined as described in Begg et al. [13] by calculating the relative movement (RM) at different time points after BrdU washout: RM = (*F*_*L*_ − *F*_G1_)/(*F*_G2/*M*_ − *F*_G1_), where *F*_*L*_ is the mean PI fluorescence of undivided BrdU-labelled cells, *F*_G1_ is the mean PI fluorescence of the G1 cells and *F*_G2/*M*_ is the mean PI fluorescence of the G2/M cells. RM values were plotted over time and after linear curve fitting, the time where RM = 1 (i.e., length of S phase) was determined.

### Statistical analysis

Statistical analysis and graphs were produced using GraphPad Prism v8.1.1 (GraphPad Software, San Diego CA). DNA fibre data are representative of three independent experiments, with >100 fibres analysed per treatment for each assay. Foci counting experiments were performed three times, with >10 randomly assigned fields captured by confocal microscopy per condition. For colony formation assays, data are representative of three independent experiments from triplicate wells, with PE and SER_10_ (survival enhancement ratio at an SF of 0.10) shown. Data were analysed by two-way ANOVA, with Bonferroni or Sidak’s post-tests used for multiple comparisons. Spearman’s rank correlation tests were used to assess homoscedasticity, and checks were performed for Gaussian distribution during analysis. Differences were considered significant at a *P* value of <0.05, and unless stated, all results are presented as mean ± SD.

## Results

### TOPK supports S phase by the protection of replication fork progression in tumour cell lines

Our previous paper reported that (1) TOPK suppression increases sensitivity to irradiation in a tumour-specific manner; (2) TOPK suppression alters G2/M checkpoint activity in tumour cells by blocking inhibitory phosphorylation of Cdk1; and (3) TOPK suppression promotes radiation-induced tumour cell death by retaining post-IR chromosomal damage and increasing the rate of multinucleation following cell division [10]. Our group and others have observed that whilst many cancers respond to TOPK treatment, a small subset is resistant. As a consequence, the question of how targeting TOPK differentially sensitised certain tumour cells to irradiation whilst sparing normal tissue remained.

Cancer cells have fundamental deficiencies in G1/S cell-cycle checkpoint control, whilst non-carcinogenic cell lines are checkpoint proficient. This differential characteristic enables unrestricted proliferation but also creates a potential (and exploitable) weakness for cancer cells on entry to, and during, replication—i.e., replication stress [14, 15]. In order to survive, many tumour subtypes have developed phenotypes, which provide protection against genomic damage arising as a consequence of replication stress [1]. Acting from this background, we questioned whether an increase in TOPK activity might be providing a protective advantage to cancer cells with unregulated replicative progression. We chose to tackle this question by analysing the replication stress response of a TOPK overexpressing cell line (H460) when placed under conditions, which disrupted the process of cellular progression through S phase, and irradiation, which activated the DNA damage response.

**Fig. 1:**
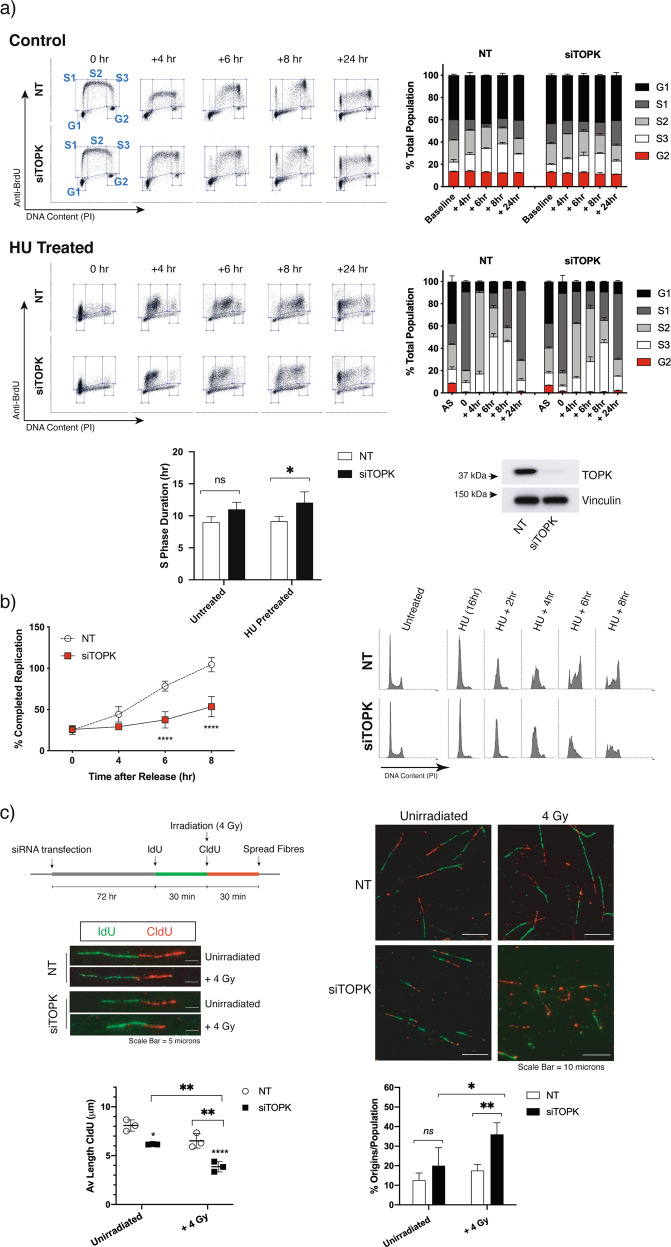
TOPK facilitates S-phase progression in cancer cell lines. **a** Analysis of S-phase progression through BrdU pulse-chase labelling. Transfected H460 cells (non-targeting (NT) and siTOPK) were untreated (top) or synchronised at the G1/S border by overnight exposure to 1 mM hydroxyurea (bottom) followed by a 30 min pulse of BrdU. Progression through S phase was determined by cytometric analysis of BrdU-positive cells. Relative fraction of BrdU negative cells in G1 (black) or G2 (red) phase, and BrdU-positive cells in early (S1: BrdU+/2N), mid (S2: BrdU+/>2N to <4N) and late (S3: BrdU+/4N) S phase is shown as a percentage of the total population at 4, 6, 8 and 24 h post-BrdU uptake. Bar graph indicates analysis of S-phase duration calculated from mean propidium iodide (PI) fluorescence in the BrdU-positive population for each condition, using two-way ANOVA with Bonferroni post-tests; ns = not significant, *p < 0.05. Immunoblot image is presented to confirm knockdown efficiency. **b** Replication restart analysis. Recovery from overnight treatment with 1 mM hydroxyurea (HU) and progression to G2 was assayed in transfected cells following release into medium containing 100 ng/ml nocodazole. Samples were analysed at 4, 6 and 8 h post-release for 4N DNA content by flow cytometry using propidium iodide (PI) staining. Results from hydroxyurea pretreated samples were standardised relative to corresponding asynchronous samples incubated in 100 ng/ml nocodazole from the time of release. Graph shows the mean 4N population from three independent experiments for each condition, using two-way ANOVA with Bonferroni post-tests; ****p < 0.0001. Representative PI plots are shown. **c** Replication fork progression using DNA fibre analysis. Control (NT) and siTOPK transfected H460 cells were sequentially labelled with IdU (30 min) followed by CldU (30 min) before harvesting for DNA fibre analysis. Irradiation (4 Gy) was performed immediately prior to washout and addition of CldU-containing medium. Average fibre length and number of new replication origins were analysed for each condition. Fibre length data were analysed by two-way ANOVA with Sidak’s multiple comparisons test (****p < 0.0001). Data are representative of three independent experiments, with >100 fibres analysed per treatment for each assay. Percentage replication origins were compared using two-way ANOVA (not significant (ns); **p < 0.01) with Sidak’s multiple comparisons post-test (*p < 0.05). Treatment schematic and representative images of DNA fibres are shown.

Whilst the role of TOPK as an enforcer of Cdk1 inhibitory phosphorylation in the late G2 phase of the cell cycle is well established [16], our investigations have also pointed to a role for TOPK during G1 exit and S-phase progression [10]. To test the effect of TOPK on S-phase regulatory control in cancer cells, we depleted TOPK in H460 lung cancer cells and tracked the process of replication over a 24-h period by pulsed BrdU labelling. This experiment revealed that the rate of progression through S phase is retarded to some extent by ablation of TOPK when compared to non-targeting (NT) controls (Supplementary Fig. S1a), although the increase in overall S-phase duration was not significant (Fig. 1a). By incubating cells overnight with 1 mM HU, we were able to induce mild replication stress by halting replication fork progression and arresting cells at the G1/S border. Here, TOPK depletion significantly reduced the rate of S-phase progression on recovery from HU treatment (Supplementary Fig. S1a), resulting in a 3 h increase in the total duration of S phase on average (Fig. 1a). To consolidate these findings, replication restart assays were used to assess the role of TOPK in recovery and completion of replication following HU-induced fork stalling. After release into fresh medium, cycling cells were captured prior to subsequent cell division with nocodazole. Using 4N DNA complement as a measure of S-phase completion, these experiments demonstrated that close to 100% of NT-transfected H460 cells released at the G1/S border had recovered from fork arrest and completed replication at the 8 h mark, yet only 50% of TOPK KD cells had fully progressed through S phase at this timepoint (Fig. 1b). These results further indicate that TOPK is not only facilitating the timely progression of cancer cells through S phase, but also supporting the resumption of replication at stalled forks.

As radiation is known to arrest replication, we wanted to assess whether radiation sensitisation in TOPK-depleted cells could be explained by enhanced recovery from radiation-induced fork stalling. To this end, we used the DNA fibre assay, the gold standard assay to monitor fork speed progression and origin firing. We treated TOPK-depleted H460 cells with IdU for 30 min, followed by 30 min of labelling with CldU immediately after exposure to 4 Gy irradiation (*n* = 4) (Fig. 1c). By comparing incorporation of these nucleotide analogues into DNA before and after irradiation, we were able to gauge whether TOPK KD influenced DNA replication after radiation. The effect of radiation on fork progression was significantly greater following TOPK depletion, with an average 50% reduction in relative fibre length in the 30 min following exposure to 4 Gy, compared to an average 25% reduction in relative fibre length measured for controls (Fig. 1c and Supplementary Fig. S1b). Additionally, firing of new origins doubled in the TOPK-ablated population following irradiation (Fig. 1c)—a finding which points to increased endogenous replication stress and altered regulatory control of the S-phase checkpoint control in these cells.

### TOPK suppression increases irradiation-induced single-stranded DNA (ssDNA) foci

**Fig. 2:**
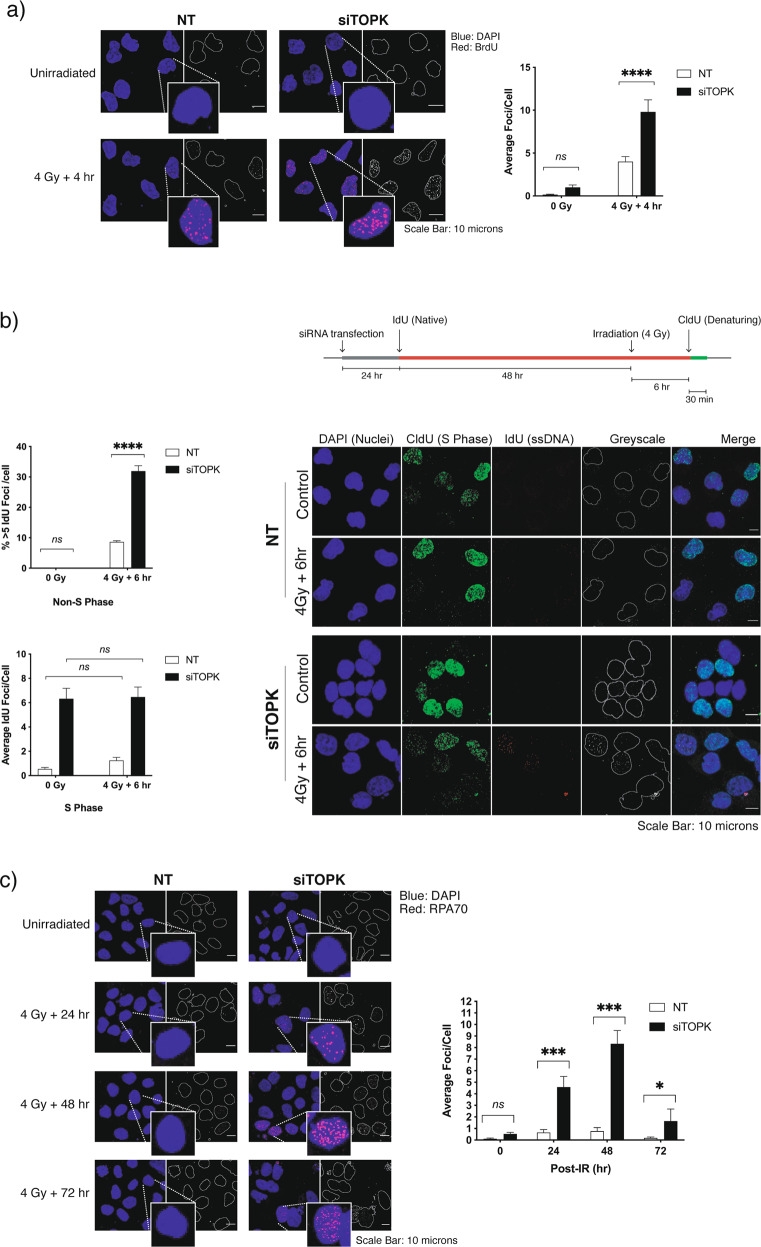
TOPK knockdown suppresses recovery from replication stress and radiation damage. **a** Radiation-induced ssDNA foci analysis. TOPK-ablated H460 cells were grown on coverslips and treated with BrdU for 48 h. Coverslips were exposed to 4 Gy irradiation, at which point the growth medium was replaced. After a 4-h recovery time, cells were fixed for immunocytochemical analysis. Slides were prepared from stained coverslips and microscopic images were assayed for presence of BrdU-positive ssDNA foci using DAPI as a counterstain. At least ten fields of view were used per condition and results were analysed by two-way ANOVA with Sidak’s multiple comparisons post-test (ns (not significant); ****p < 0.0001). Scale bar = 10 μm. **b** Radiation-induced ssDNA foci analysis with discrimination between non-S and S-phase cells. Coverslips bearing non-targeting siRNA (NT) or TOPK-ablated H460 cells were pretreated with IdU prior to 4 Gy irradiation followed by 30 min CldU incubation as indicated by the treatment schematic. Coverslips were stained for non-denatured ssDNA foci (ldU, red) prior to re-staining for CldU-positive S-phase cells (green) after fixing and denaturing DNA by brief exposure to DNase (30 min, 10U). Images were captured and assayed for ssDNA foci in non-replicating nuclei by excluding CldU-positive S-phase nuclei from analysis. Representative images are shown. Each experiment was performed three times, and >10 randomly assigned fields were captured by confocal microscopy per condition. Non-S-phase graphs represent percentage of cells bearing >5 ssDNA foci/nucleus for each condition, and S-phase graphs represent average foci/cell. Results were analysed by two-way ANOVA (ns (not significant)) with Sidak’s multiple comparisons post-tests (****p < 0.0001). Scale bar = 10 μm. **c** Accumulation of RPA70 foci in TOPK-ablated cells after irradiation. H460 cells were siRNA transfected and plated on coverslips and fixed at 24, 48 and 72 h following irradiation (4 Gy). Slides were stained for RPA70, and images from ten randomly assigned fields were captured by confocal microscopy per condition. Images were analysed for presence of RPA foci using DAPI as a nuclear counterstain. Results were analysed by two-way ANOVA with Sidak’s multiple comparisons post-tests (ns (not significant); *p < 0.05; ***p < 0.001). Scale bar = 10 μm.

Replication fork stalling and 5′-3′ DNA-end resection during double-strand break (DSB) repair can lead to the exposure of single stranded (ssDNA) [17, 18]. To assess whether TOPK depletion affects the accumulation of ssDNA, we incubated cells with BrdU for 48 h to allow genomic incorporation for all cells prior to irradiation and stained fixed cells with anti-BrdU under non-denaturing conditions to identify ssDNA regions produced during recovery from irradiation damage. Indeed, nuclei of TOPK-depleted cells retained more than twice as many BrdU foci on average when compared to those with non-targeting siRNA at 4 h following irradiation (4 Gy) (Fig. 2a).

To investigate only the ssDNA foci arising from resection during DSB repair (cells predominantly in G2), S-phase cells were excluded from analysis. H460 cells were cultured with IdU-containing media to allow subsequent detection of focal nuclear regions of ssDNA, and DNA was labelled with CldU in the 30 min prior to harvest, to identify cells in S phase. TOPK depletion resulted in more radiation-induced ssDNA foci in non-S-phase cells (Fig. 2b), suggesting that TOPK depletion leads to more DNA resection. TOPK-depleted cells also produced five to six times more ssDNA foci during S phase, indicative of increased replication fork stalling in TOPK-depleted cells with and without exogenous DNA damage (Fig. 2b). Together these results suggest that TOPK plays a role during S phase, enabling cells to cope with replication stress.

Regions of ssDNA generated during replication fork collapse and IR-induced DNA damage are stabilised by the RPA complex, which, alongside Rad52, enables Rad51 filament formation [19, 20]. Using RPA70 (RPA 1) as a marker for post-IR ssDNA, the impact of TOPK depletion on RPA foci formation and resolution in irradiated H460 cells was quantified by confocal microscopy. The kinetics of RPA foci formation following 4 Gy IR exposure was significantly altered by TOPK KD, with a doubling of average foci/cell at 6 h post-IR and failed resolution at 24 h (Supplementary Fig. S2a). In fact, RPA foci were still retained in TOPK-suppressed cells after 48 h or even at 72 h after irradiation (Fig. 2c).

Protection of ssDNA by RPA binding is critical to the maintenance of genome stability. Conditions that impair RPA recruitment or that exhaust the available RPA pool leave regions of exposed ssDNA vulnerable to breakage and the generation of single-strand break and DSB [21, 22]. Data from these experiments indicate that TOPK suppression causes a greater degree of intrinsic replication damage, but that it is the retention of irradiation-induced damage in combination with reduced tolerance for replication stress, which is contributing to radiosensitisation.

### TOPK promotes radioresistance via S-phase-mediated DNA damage signalling

**Fig. 3:**
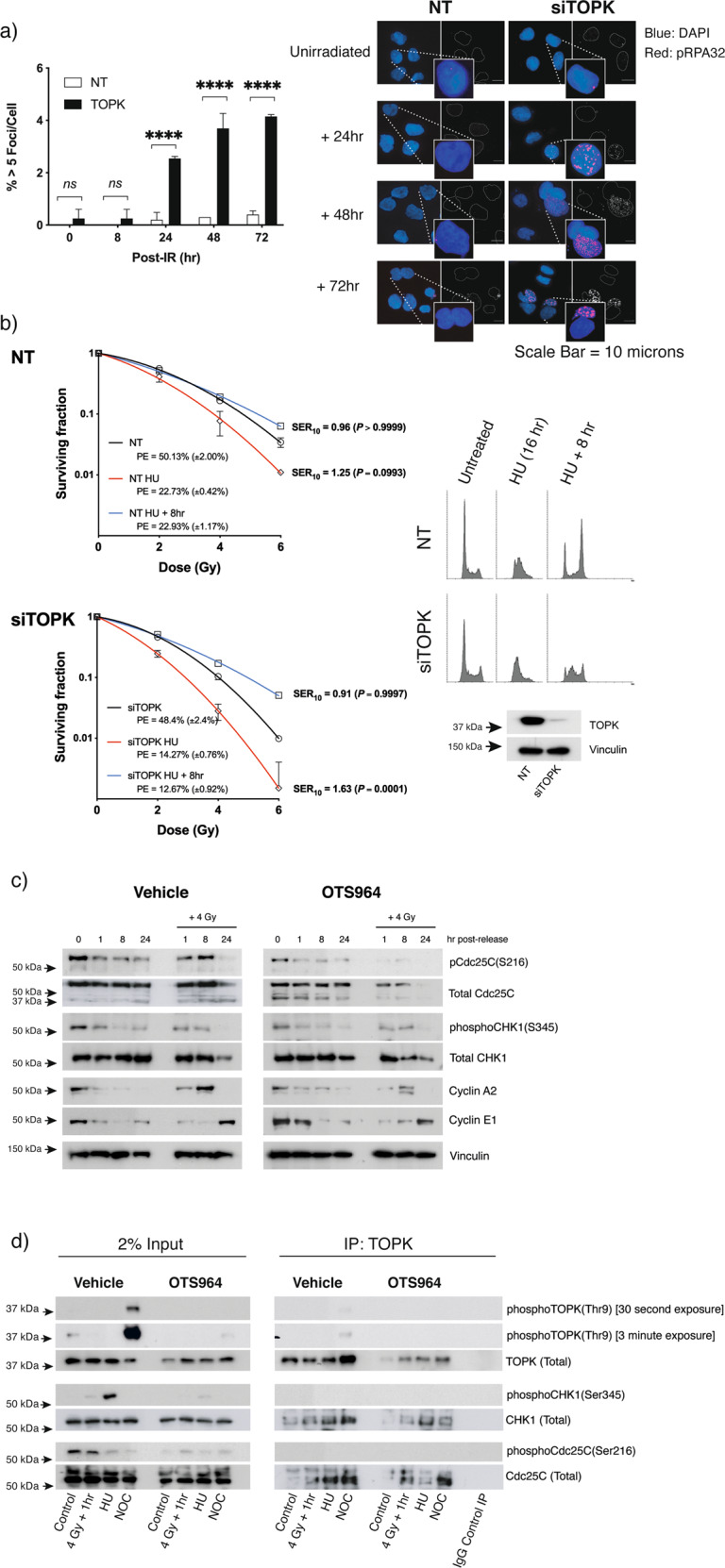
TOPK modulates radiosensitivity by interaction with replication checkpoint signalling intermediates. **a** Accumulation of pRPA32 foci in TOPK-depleted cells after irradiation. TOPK-ablated H460 cells were plated on coverslips and fixed at 0, 8, 24, 48 or 72 h following irradiation (4 Gy). Slides were stained for RPA32 phosphorylated at serine residues 4 and 8, and images from ten randomly assigned fields were captured by confocal microscopy per condition. Images were analysed for presence of foci using DAPI as a nuclear counterstain. Percentage of nuclei with >5 foci for each timepoint was analysed by two-way ANOVA with Sidak’s multiple comparisons post-tests (**p < 0.01; ****p < 0.0001) and is representative of two independently performed experiments. Error bar = SEM. **b** Effect of TOPK depletion and replication stress on post-irradiation survival. TOPK was transiently knocked down with siRNA (siTOPK) in H460 cells. After replating, cells were exposed to hydroxyurea for 16 h, then irradiated immediately, or at a timepoint 8 h following release. Colonies were stained after 14 days’ growth and analysed for the effect of replication stress on radiation sensitivity by factorial two-way ANOVA. Survival curves were fitted using non-linear regression. Data are representative of three independent experiments and are presented as mean ± standard deviation (SD) from triplicate wells. Plating efficiency (PE) and SER_10_ (survival enhancement ratio compared to NT controls at a surviving fraction of 0.10) are indicated. Propidium Iodide (PI) cytometry plots of samples taken at the time of irradiation are shown to indicate cell-cycle distribution. Immunoblot image is presented to confirm knockdown efficiency. **c** Suppression of Cdc25c and CHK1 phosphorylation by the TOPK inhibitor OTS964. H460 cells were synchronised at the G1/S border by double thymidine block and treated with OTS964 (20 nM) for 16 h. Following exposure to 4 Gy irradiation, cells were released into fresh growth medium, and protein lysates prepared for analysis of phosphorylation status of Cdc25C and CHK1 at 1, 8 and 24 h post-release. Vinculin was used to standardise protein loading, and immunoblots of Cyclin A2 and Cyclin E1 were used to confirm cell-cycle progression. Representative immunoblots shown (*n* = 3). **d** Immunoprecipitation showing interaction between TOPK and CHK1 and Cdc25c. TOPK-bound proteins were extracted from H460 cell lysates following OTS964 treatment and/or irradiation (4 Gy), overnight treatment with hydroxyurea (HU, 1 mM) or Nocodazole (NOC, 100 ng/ml) by immunoprecipitation. Pull-down using mouse IgG was used as negative control. Samples were probed for Cdc25C and CHK1 co-immunoprecipitates by western blotting. Immunoblots are representative of three independent experiments, with 2% input lysate shown for comparison.

In the presence of IR-induced DNA damage, RPA binding recruits ATR to DNA lesions and promotes RPA32 phosphorylation at serine residues 4 and 8 [23]. RPA32 phosphorylation marks the culmination of sequential phosphorylation by ATR, ATM and DNA-PK [24, 25], and is widely regarded a marker for replication checkpoint activation in response to DNA damage [20]. Our observation of a small, but consistent increase in the population of TOPK-suppressed cells with >5 pRPA32 (S4/S8) foci in the 24–72-h period after irradiation (Fig. 3a) supports evidence of functional DNA repair activity, and corresponds with the increase in total RPA foci seen in Fig. 2c. Localisation of hyperphosphorylated RPA32 foci to stalled and collapsed replication forks is known to directly correlate with DSBs formed as a consequence of DNA damage-induced replication stress [25]. Unlike DSBs directly attributable to IR, these lesions which comprise an accumulation of secondary DSBs formed during replication and in association with HR, are irreparable, and lead to cellular senescence [26]. Furthermore, when TOPK-depleted cells were subjected to increased replication stress (by HU pretreatment), irradiation produced a marked increase in the presence of γH2AX foci during the 24-h post-IR period when compared to NT-transfected cells (Supplementary Fig. S2b). TOPK depletion in combination with HU treatment was also associated with an increase in cells displaying diffuse pan-nuclear γH2AX staining. Nuclear-wide formation of γH2AX occurs during catastrophic replication stress, and invariably precedes irreversible cell death [27].

RPA32 phosphorylation stimulates G2/M checkpoint arrest via CHK1 [24]. ATR-mediated activation of CHK1 at serines 317 and 345 triggers a signalling cascade which regulates S-phase progression and G2/M checkpoint activation through inhibitory interactions with Cdc25 family proteins and Cdk1 and 2 [28, 29]. Failure to mount an effective G2/M checkpoint response increases unresolved DSBs and γH2AX foci as a consequence of DNA damage and replicative stress [30], which causes chromatid exchanges and triggers mitotic catastrophe. Hence from a mechanistic standpoint, we reasoned that the higher degree of DNA damage foci retained in TOPK-depleted cells when exposed to replication stressors and/or irradiation damage might be linked to changes in activity and expression of S-phase checkpoint signalling intermediates downstream of ATR. In control cells, HU treatment stimulated CHK1 phosphorylation at Ser345, and activated the G2/M checkpoint within 8–24 h of release (phospho-Cdc25C Ser216) (Supplementary Fig. S3). Suppression of TOPK weakened phosphorylation of both CHK1 and Cdc25C after exposure to HU alone and when combined IR, indicating that S-phase and G2M checkpoints are impaired for proliferating cancer cells in the absence of TOPK (Supplementary Fig. S3).

Considering the delayed and persistent nature of post-IR DNA damage measured in TOPK-depleted cells, we next considered the relationship between clonogenicity, TOPK expression and radiation resistance under conditions of replication stress. Here, colony formation assays were performed on cells, which were irradiated whilst in a state of HU-induced replication stress, or following an 8-h period of recovery. Irradiation of cells under conditions of increased replication stress enhanced radiation sensitivity for either NT- or TOPK-transfected H460 cells (SER_10_^NT^ = 1.25), with greater radiosensitisation observed with TOPK KD (SER_10_^TOPK^ = 1.63). For cells given 8 h of recovery time prior to irradiation, the radiosensitising effect of HU pretreatment was lost (SER_10_^NT^ = 0.96, SER_10_^TOPK^ = 0.91) (Fig. 3b). This experiment demonstrated clearly the relationship between TOPK expression, sensitivity to radiation-induced DNA damage and replicative stress in the H460 cell line.

To further assess the role of TOPK kinase activity on checkpoint signalling, we used pharmacological inhibition, rather than ablation, for subsequent experiments. Dose-response and colony formation assay experiments demonstrate that the TOPK inhibitor compounds OTS964 and OTS514 both sensitise H460 cells to irradiation within the nanomolar range (Supplementary Fig. S4). A decision was made to use OTS964 exclusively for this study due to its more attractive pharmacokinetic profile for in vivo work.

Cells were synchronised at the G1/S border by double thymidine block and TOPK was inhibited with OTS964. On release, TOPK-inhibited cell lysates showed significant suppression of phospho-Cdc25C, and partial reduction of CHK1 phosphorylation (Fig. 3c). This downregulation of CHK1 and Cdc25C phosphorylation also occurred in TOPK-ablated cells after G1/S synchronisation/release using HU (Supplementary Fig. S3). Furthermore, TOPK co-immunoprecipitates with Cdc25C and CHK1 (Fig. 3d). This interaction was observed in untreated controls as well as in cells following irradiation-induced DNA damage, populations synchronised in G1/S by HU, or those synchronised in G2/M by nocodazole treatment. Additionally, synchronised H460 cells did not demonstrate any significant change in activation of upstream DNA damage response intermediates (ATM autophosphorylation, ATR phosphorylation or DNA-PKcs phospho-activation) with TOPK KD following irradiation. Nor did we detect any co-immunoprecipitation between TOPK and ATM, ATR or DNA-PKcs before, or after OTS964 treatment (data not shown).

In light of these results, our experiments support a role for TOPK as a mediator of S-phase progression and post-replication responses via CHK1 and Cdc25C phospho-activation. These data imply that not only does TOPK facilitate mitotic progression at the G2/M checkpoint via Cdk1, but TOPK is also in response to replication stressors (such as HU treatment or irradiation) by influencing the action of key intermediates such as CHK1.

### TOPK inhibition potentiates fractionated radiotherapy in vivo

**Fig. 4:**
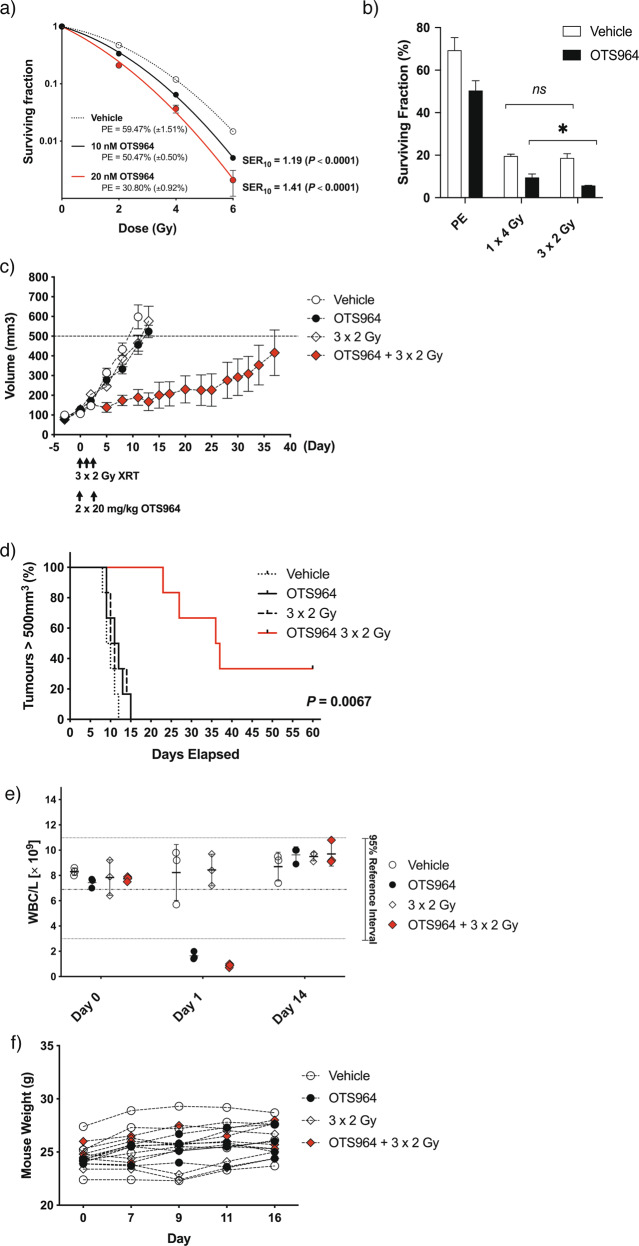
TOPK inhibition enhances tumour sensitivity to fractionated irradiation. **a** In vitro radiosensitisation by OTS964 in H460 lung cancer cells using a single radiation dose. Cells were plated at known densities in six-well plates, pretreated with 10 or 20 nM OTS964 for 1–3 h prior to irradiation, and incubated at 37 °C. Growth medium was replaced after cells had been exposed to OTS964 for a total of 24 h, and colony formation assessed after 12 days’ growth. Survival curves were fitted using non-linear regression. Data are representative of three independent experiments and are presented as mean ± standard deviation (SD) from triplicate wells. Plating efficiency (PE) and SER_10_ (survival enhancement ratio at a surviving fraction of 0.10) are indicated. **b** In vitro radiosensitisation by OTS964 using single or fractionated irradiation. TOPK was inhibited in H460 cells using 20 nM OTS964 and cells were exposed to irradiation in a single dose (4 Gy) or fractionated dosing (3 × 2 Gy, delivered at 24 h intervals). Colony formation was assessed after 14 days’ growth. Plating efficiency (PE) was calculated from unirradiated colonies for each condition, and the surviving fraction of colonies after irradiation for each treatment group was analysed using two-way ANOVA with Bonferroni post-tests (ns (not significant); *p < 0.05). **c** Tumour growth delay in xenografts treated with OTS964 and fractionated radiation. CD-1 nude mice bearing H460 tumours were treated with OTS964 (20 mg/kg; days 0 and 2) and/or irradiation (XRT, 2 Gy; days 0, 1, 2; *n* = 6). Tumour volume was measured twice-weekly over a 40-day period. Growth delay data are presented as average tumour volume. Time for tumours to reach 500 mm^3^ was analysed by log-rank survival tests (*p* = 0.0067; panel (**d**)). Mice were monitored during the 16-day post-treatment period for blood leucocyte density on days 0, 1 and 14 (white blood cells; WBC) (panel (**e**); *n* = 3 per treatment group), and bodyweight (panel (**f**)).

To expand on our in vitro findings, and to investigate the preclinical validity of TOPK as a therapeutic target, we used in vivo studies to test the efficacy of OTS964 on subcutaneous xenografts in combination with radiation therapy. To assess the radiosensitisation efficacy of the OTS964 compound, we performed colony formation assays in H460 and Calu-6 lung cancer cell lines. Both cell lines showed significant radiosensitisation using a 24 h incubation with 20 nM OTS964 (Fig. 4a and Supplementary Fig. S5a). A fractionated dosing strategy for irradiation treatment was found to improve radiosensitisation by OTS964 at this concentration (Fig. 4b), and so this fractionated radiation regime was adopted for in vivo studies.

For growth delay experiments, CD-1 nude mice were inoculated subcutaneously with H460 cells and when xenografts reached 80 mm^3^, tumours were irradiated at 2 Gy for 3 consecutive days. OTS964 was administered at 20 mg/kg by intraperitoneal injection 1 h prior to irradiation on days 0 and 2. Tumour growth was almost completely suppressed for 20–30 days in the group which had undergone combined drug/irradiation treatment; whilst those which received OTS964 or fractionated irradiation alone had tumour growth comparable to the vehicle only treated group, reaching a volume of 500 mm^3^ within 10 days (*n* = 6 for each condition) (Fig. 4c). Of the mice in the combination group, two had tumours which failed to reach 500 mm^3^ during the course of the experiment (Fig. 4d; *P* = 0.0067). A comparable delay in tumour growth was also observed combining fractionated irradiation with OTS964 in Calu-6 lung cancer xenografts (Supplementary Fig. S5b).

Weight loss was not observed in CD-1 nude mice exposed to OTS964 at 20 mg/kg (Fig. 4f), however, analysis of blood samples showed a significantly lower total white blood cell (WBC) count 24 h following administration (Fig. 4e). Daily analysis of WBC count revealed complete recovery had occurred 8–10 days following the onset of treatment (Supplementary Fig. S5c). Treatment-associated leukocytopenia was primarily associated with suppression of the relative lymphocyte and granulocyte populations, which had resolved 2 weeks later (Supplementary Fig. S5d). This is most likely to reflect the previously documented influence of OTS964 on haematopoietic stem cell differentiation to CD41+ megakaryocytes, with a concomitant suppression of alternative lineages [31]. Although the fractionated irradiation schedule produced a small, but significant shift in the relative lymphocyte and granulocyte populations, this was not reflected in the total WBC count, and did not produce any additive effect when combined with OTS964 treatment.

Our in vivo findings indicate that inhibition of TOPK provides a synergistic radiosensitising effect on tumour cell death, with transient leukocytopenia resulting from treatment. The conclusion from these observations is that systemic administration of OTS964 preferentially affects tissues with high proliferative turnover, increasing efficacy for treatment strategies which specifically deliver DNA-damaging agents to the tumour.

## Discussion

Carcinogenic transformation promotes mutational change that confers a protective advantage, or resistance, to genomic damage occurring during replication—hence the recent interest in potential drug candidates that interfere with replication and/or enhance replication stress [15]. This study provides evidence indicating that TOPK is modulating regulatory control over cell-cycle progression through events occurring in S phase. The impact of TOPK suppression is particularly prescient to cancer cells, which are generally exposed to higher replication-associated stress than surrounding normal tissue. This would also account for the reduced proliferative turnover of cancer cells measured by our lab and others after TOPK depletion or inhibition.

Our results show that TOPK interacts with the CHK1 and Cdc25c complex, key players in the replication damage-induced checkpoint. We showed that, in irradiated TOPK-depleted cells, replication fork speed slows, origin firing increases and more regions of ssDNA are formed compared to TOPK-proficient cells. These responses caused an accumulation of unresolved RPA and γH2AX foci, which remain at 72 h after IR. This persistence of RPA foci at late phase post-IR time points indicates that (1) the process of post-damage replication stress is ongoing for TOPK-ablated cells, and (2) that resolution of single-stranded DNA in the genome is impaired. Removal of TOPK-mediated regulatory control precipitates a series of events which culminate in a higher susceptibility to exogenous DNA damage. As such, TOPK suppression in combination radiotherapy has the potential to induce production of post-replicative DSBs [32]. Although the nature of the interaction between TOPK, CHK1 and Cdc25C remains elusive, our observations strongly support a model in which upregulation of TOPK in tumour cells plays a vital function during the increased cellular demands of replication.

Our in vivo experiments were able to demonstrate a significant delay in tumour growth by OTS964-mediated TOPK inhibition when used in combination with XRT. OTS964 has previously been established as a single-agent anti-cancer drug for a treating variety of cancer subtypes in vitro, and in vivo when delivered to tumour-bearing mice at 40 mg/kg (intravenously, bi-weekly for 18 days) or 50–100 mg/kg (oral dosing, daily for 2 weeks) [31]. However, the high doses of OTS964 used in these studies were associated with adverse effects. Our study has demonstrated that when used in combination with fractionated radiation therapy, a small overall dose of OTS964 (2 × 20 mg/kg) was able to suppress tumour growth. Although we did see leukocytopenia immediately following exposure, this adverse effect of OTS964 was transient, with minimal recovery time afforded by the shorter dosing requirements demonstrated in our study.

Lin et al. [33] have reported that PBK/TOPK is not an essential gene for cancer cell survival and that off-target inhibition of CDK11 by OTS964 contributes to the anti-proliferation effect of the compound on cancer cell lines. We have previously demonstrated the non-essential nature of TOPK for cancer cell survival by using a CRISPR knockout to create a TOPK-null stable HAP1 cell line [10]. Off-target inhibition of kinases by OTS964 has been previously reported [31]. Our conclusions regarding the impact of TOPK on radiation sensitivity and replication stress via interactions with CHK1 have been based on concomitant use of siRNA KD experiments as well as through inhibition with OTS964. We have verified the efficacy of OTS964 as an inhibitor of TOPK on our cell lines using its substrate motif as a marker (Supplementary Fig. S4c). Importantly, this enabled us to titrate the concentration of OTS964 used in our investigations to an effective dose lower than the KD of CDK11 for OTS964 binding, but within the working range for TOPK activity.

Targeting key checkpoint kinases that regulate DNA replication and repair is seen as a means to introduce tumour-specific vulnerabilities, which can improve the therapeutic window for standard treatment modalities [34]. CHK1 inhibition, in particular, is known to slow global replication rates [35], trigger origin firing [36], increase the phosphorylation of ATR targets p53, SMC1 and RPA (which increases SSBs and DSBs) [37], suppress homologous recombination repair [38] and promote premature mitotic entry through inappropriate Aurora Kinase activation [39]. However, as CHK1 is expressed by cancer and normal cells, early clinical trials with CHK1 inhibitors have been associated with dose-limiting side effects [40–42]. Our results show that TOPK inhibition suppresses CHK1-Cdc25C signalling but, unlike CHK1 inhibition, the likelihood of toxicity and adverse effects to normal tissue when targeting TOPK is reduced, as TOPK is only limitedly expressed in normal tissues.

This paper presents evidence that TOPK plays a supporting role in CHK1-mediated maintenance of DNA replication fidelity, and that suppression of TOPK activity and/or expression disrupts the process of replication such that cancer cells are rendered increasingly vulnerable to radiation damage. Our conclusion is that increased TOPK expression provides a competitive survival advantage during carcinogenic transformation by enabling proliferating cells to tolerate oncogene-induced replication stress. As a potential anti-cancer target, TOPK would appear to be selective for highly proliferative cancers with an increased level of endogenous replication stress, with the efficacy of TOPK inhibitory treatment being enhanced by delivery in combination with genotoxic agents. This finding is particularly relevant in light of our emerging understanding of acquired resistance to replication-associated stress as an oncogenic survival mechanism.

## Supplementary information

Supplementary Information

Supplementary Figure S1

Supplementary Figure S2

Supplementary Figure S3

Supplementary Figure S4

Supplementary Figure S5
