# Supplementary material for: Targeting TOPK sensitises tumour cells to radiation-induced damage by enhancing replication stress
Source: Cell Death Differ. 2020 Nov 9;28(4):1333–46. doi: 10.1038/s41418-020-00655-1 (PMC8027845; doi:10.1038/s41418-020-00655-1)
Supplement: Supplementary file 1 — Supplementary Information [file 41418_2020_655_MOESM1_ESM.docx]

**Supplementary Materials and Methods**

*Drug Treatment and Irradiation*

OTS964 and OTS514 (OncoTherapy Science, Inc.) were dissolved in DMSO and used at concentrations stated. Hydroxyurea (HU) was dissolved in water and used at a concentration of 1 mM unless otherwise stated. Irradiation for *in vitro* experiments was delivered at a dose rate of 1.938 Gy × min^−1^ using a GSR D1 caesium-137 irradiator (Gamma Service).

*Immunoblotting*

Cell lysates were prepared in RIPA lysis buffer (Thermo Scientific) with protease inhibitors (Roche) and phosphatase inhibitors (Sigma) and protein concentration was determined using the BCA assay (Thermo Scientific). Proteins were separated by SDS-PAGE and transferred to PVDF membranes, which were probed by overnight incubation at 4°C in primary antibody solution. Targets were detected via HRP-conjugated secondary antibodies exposed to chemiluminescence reagent (Millipore).

*Fluorescence Microscopy*

Cells were grown on coverslips at subconfluent densities and treatments were initiated during logarithmic growth phases. At the relevant timepoints, nonchromatin bound nuclear protein was removed by preincubation of coverslips with extraction buffer for 10 min on ice, after which samples were fixed with 4% paraformaldehyde for 15 min. Coverslips were blocked before incubation with primary antibody for 3 hr, followed by the corresponding fluorescent secondary antibodies for 1 hr at room temperature. Nuclei were counterstained with 0.5 μg/ml 4′,6-diamidino-2-phenylindole (DAPI), and coverslips were mounted using Vectashield medium (Vector Laboratories). Z stack images were randomly acquired under identical parameters with a Zeiss LSM710 fluorescence microscope (Zeiss) using a 63× objective for γH2AX, 53BP1 and RPA foci assessment (>10 fields per sample). Nuclear 53BP1, γH2AX and RPA foci number were analysed using ImageJ software.

*In Vivo Experiments*

Calu-6 (1× 10^6^) cells were inoculated subcutaneously with matrigel (BD Biosciences) in CD-1 nude female mice at age 55–70 days. Animals were randomly assigned to each treatment group and treatments commenced once xenografts had reached 100 mm^3^. Mice were treated with Vehicle (5% DMSO/40% PEG400) or 20 mg/kg OTS964 via intraperitoneal injection on days 0 and 2. For the radiation treatments, fractionated doses of 2 Gy/dose were delivered to tumours on days 0, 1 and 2 with a Gulmay-320 cabinet irradiator (Gulmay) at a dose rate of 2.0 Gy min^-1^, using lead shielding. The project licence covering the animal work (PPL30/3395) was approved by the Oxford University Animal Welfare and Ethical Review Body (AWERB) and granted by the UK Home Office Animals in Science Regulation Unit (ASRU) under the Animals (Scientific Procedures) Act 1986 (ASPA)).

*Statistical Analysis*

Statistical analysis and graphs were produced using GraphPad Prism v8.1.1 (GraphPad Software, San Diego CA). All statistical tests were performed as two-tailed tests and differences were considered significant at a P-value of less than 0.05. Survival curves were fitted using nonlinear regression and were analysed by factorial two-way ANOVA. Unless stated, all results are presented as mean ± standard deviation (SD).

**Supplementary Figures**

*Figure S1: TOPK expression supports S phase progression in the H460 lung cancer cell line*

a) siRNA transfected H460 cells [Non-Targeting (NT), siTOPK] were asynchronous (Control) or synchronised at the G1/S border (HU Treated) by overnight exposure to 1 mM hydroxyurea (HU). BrdU pulse-labelled cells were sampled at 4, 6, 8, and 24 hours following release into fresh medium. Proportion of cells in early S phase (S1), mid-S phase (S2) or late S phase (S3) at each timepoint is shown in graphical format, and represents results from 3 independent experiments. Data were analysed by 2-way ANOVA with Bonferroni post-tests, with significant p values indicated.

Panel b): DNA fibre ratio profile for siNT and siTOPK transfected H460 cells prepared using fibre length data collected 30 minutes before and after irradiation (4Gy). Three independent experiments were performed, with >100 fibres analysed per condition in each case. Distribution analysis of CldU/IdU ratio results for each condition are presented as a percentage of total replication events and are plotted in histogram format. Representative graph is shown.

*Figure S2: TOPK depletion alters DNA damage repair kinetics in H460 cells following irradiation*

TOPK ablated H460 cells were plated on coverslips and fixed at various timepoints in the 24 hr period following irradiation (4 Gy). Slides were stained for RPA70 (Panel a) and γH2AX (Panel b). In the case of Panel b, cells were treated with hydroxyurea for 16 hr prior to irradiation. Images from 10 randomly assigned fields were captured by confocal microscopy per condition and foci were analysed using DAPI as a nuclear counterstain. Average number of foci/nucleus for each timepoint was plotted using non-linear curve fitting, and is representative of 2 independently performed experiments. Statistical significance was determined using two-tailed nonparametric Spearman correlation. Error bars = SD. Scale bar = 10 μm

*Figure S3: TOPK suppression impairs DNA damage signalling in cancer cells subjected to replication stress.*

TOPK knockdown was achieved with siRNA and cells were exposed to 1 mM hydroxyurea for 16 hr. Following exposure to 4 Gy irradiation, cells were sampled for analysis of Cdc25C and CHK1 phosphorylation status, at 1, 6, 8, and 24 hours post-release. Vinculin was used to standardise protein loading, Cyclin E1 to confirm G1/early S phase arrest and release, and TOPK immunoblotting used to verify knockdown efficiency. Representative immunoblots shown (*n*=3).

*Figure S4: Overnight exposure to OTS964 or OTS514 suppresses TOPK-mediated phospho-signalling and tumour cell survival in a dose-dependent manner.*

a) H460 cells were cultured with varying concentrations of the TOPK inhibitor OTS964. Cell survival after 72 hour exposure was determined by resazurin reduction, and the IC_50_ for OTS964 determined after nonlinear curve fitting. Error bars = SD.

b) H460 cells were plated in 96 well plates and treated with a dose range of OTS964 (0-200 nM). Cells were exposed to 6 Gy irradiation 1 hour after addition of the TOPK inhibitor, and growth medium replaced 24 hours later. Clonogenic survival was analysed at 10 days (0 Gy) or 12 days (6 Gy) post-irradiation. Surviving fraction for each dose was plotted using nonlinear curve fitting. Graph indicates the average of 3 replicate wells (error bars = SD), and is representative of 2 independent experiments.

c/d) H460 cells were cultured with the TOPK inhibitor OTS964 (c); or OTS514 (d) and treated with Nocodazole overnight. Cell lysates were immunostained for phosphorylation of the TOPK-targeting motif [HpTGEKP] and autophosphorylation of TOPK [pTOPK(Thr9)]. Vinculin staining was used to standardise protein loading.

e) H460 cells were cultured in 96 well plates and treated with a 2-fold dilution series of OTS514. Cell viability assayed after 72 hours by resazurin reduction. The IC_50_ was determined by nonlinear curve fitting after normalising readings relative to the vehicle control. Graph indicates the average of 4 replicate wells, error bars = SD.

f/g) Cells were plated in 24 well plates, treated with OTS514 at 1 hour prior to irradiation, and removed after 18 hours. Clonogenic survival was analysed 11 days later. Plating efficiency for unirradiated colonies at each dose was measured relative to the vehicle control (f). Surviving fraction for colonies treated with vehicle control, 5 nM, and 20 nM OTS514 after irradiation was plotted using nonlinear curve fitting (g). Graph indicates the average of 3 replicate wells (error bars = SD). The SER_10_ value for 20 nM is indicated.

*Figure S5:*

a): Calu-6 cells were plated in 6 well plates, pretreated with 10 nM, 20 nM OTS964 for 2 hours prior to irradiation, and incubated at 37°C. Growth medium was replaced after cells had been exposed to OTS964 for a total of 24 hr, and colony formation assessed after 14 days’ growth. Survival curves were fitted using non-linear regression and analysed by factorial 2-way ANOVA. Data are representative of three independent experiments and are presented as mean ± standard deviation (SD) from triplicate wells. SER_10_ = survival enhancement ratio at a surviving fraction of 0.10.

b/c) Calu-6 xenografts in CD-1 nude mice were subjected to fractionated irradiation (3 x 2 Gy; n = 3) and OTS964 treatment. Tumour volume was measured over a 20 day period following onset of treatment (Panel b). Peripheral blood was harvested each day during the 14 day period following treatment onset (n = 3). Blood cells were prepared to generate a total white blood cell count (WBC; Panel c), or analysed by cytometry for relative leukocyte subpopulations (Panel d). Reference interval courtesy of Charles River.
