# Supplementary figures and images for: Targeting TOPK sensitises tumour cells to radiation-induced damage by enhancing replication stress
Source: Cell Death Differ. 2020 Nov 9;28(4):1333–46. doi: 10.1038/s41418-020-00655-1 (PMC8027845; doi:10.1038/s41418-020-00655-1)

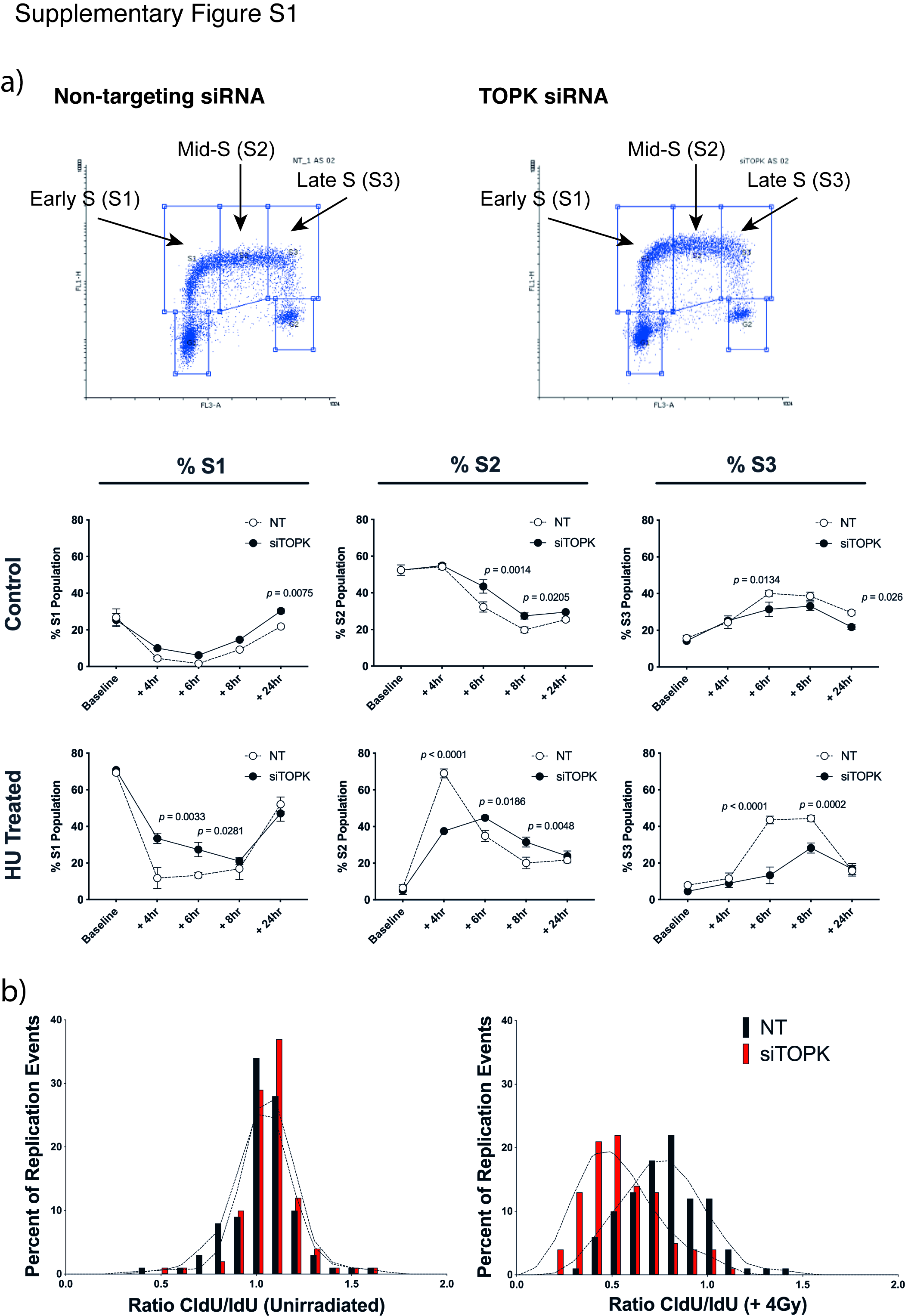

Supplement: Supplementary file 2 — Supplementary Figure S1 [file 41418_2020_655_MOESM2_ESM.tif]

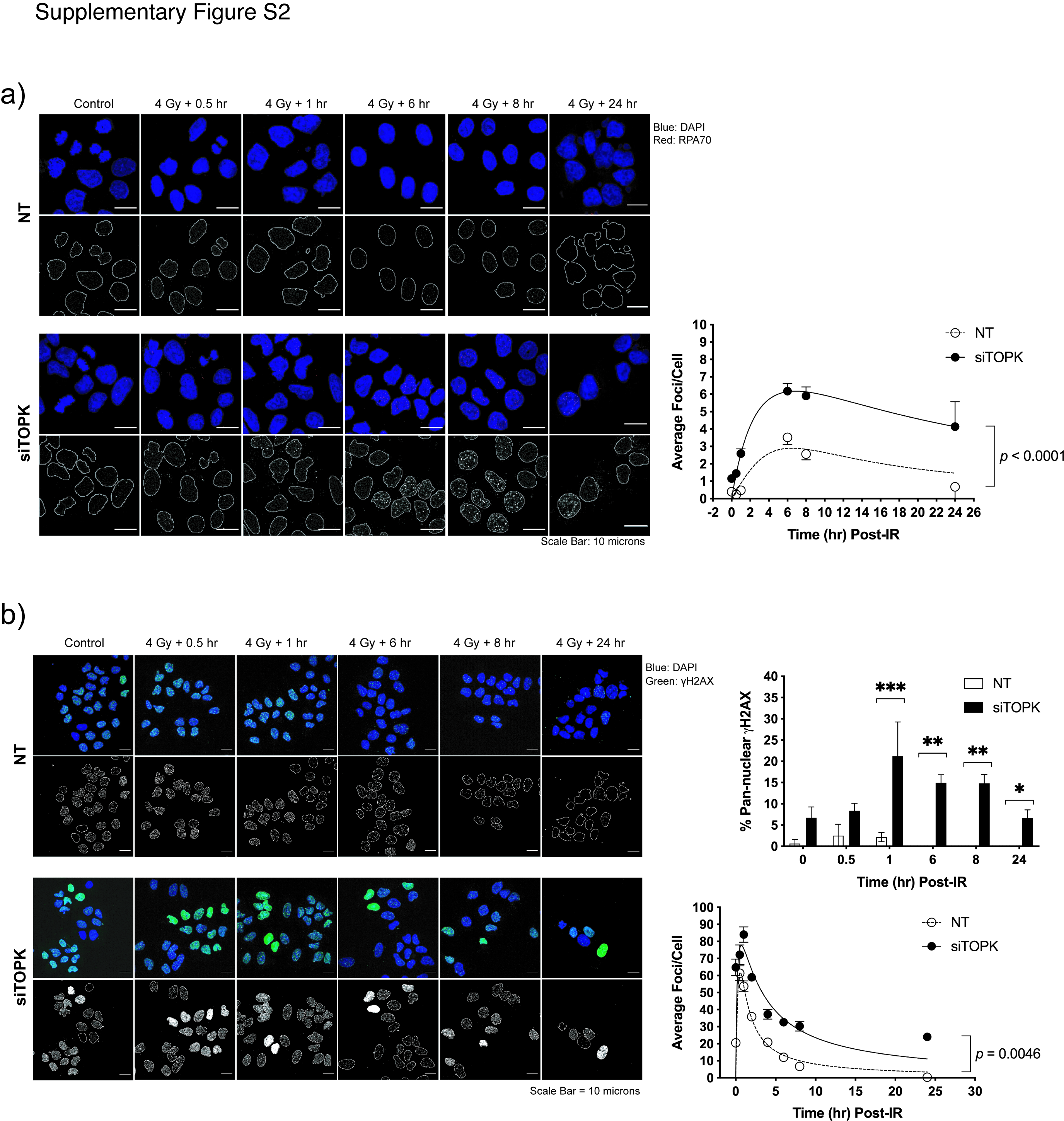

Supplement: Supplementary file 3 — Supplementary Figure S2 [file 41418_2020_655_MOESM3_ESM.tif]

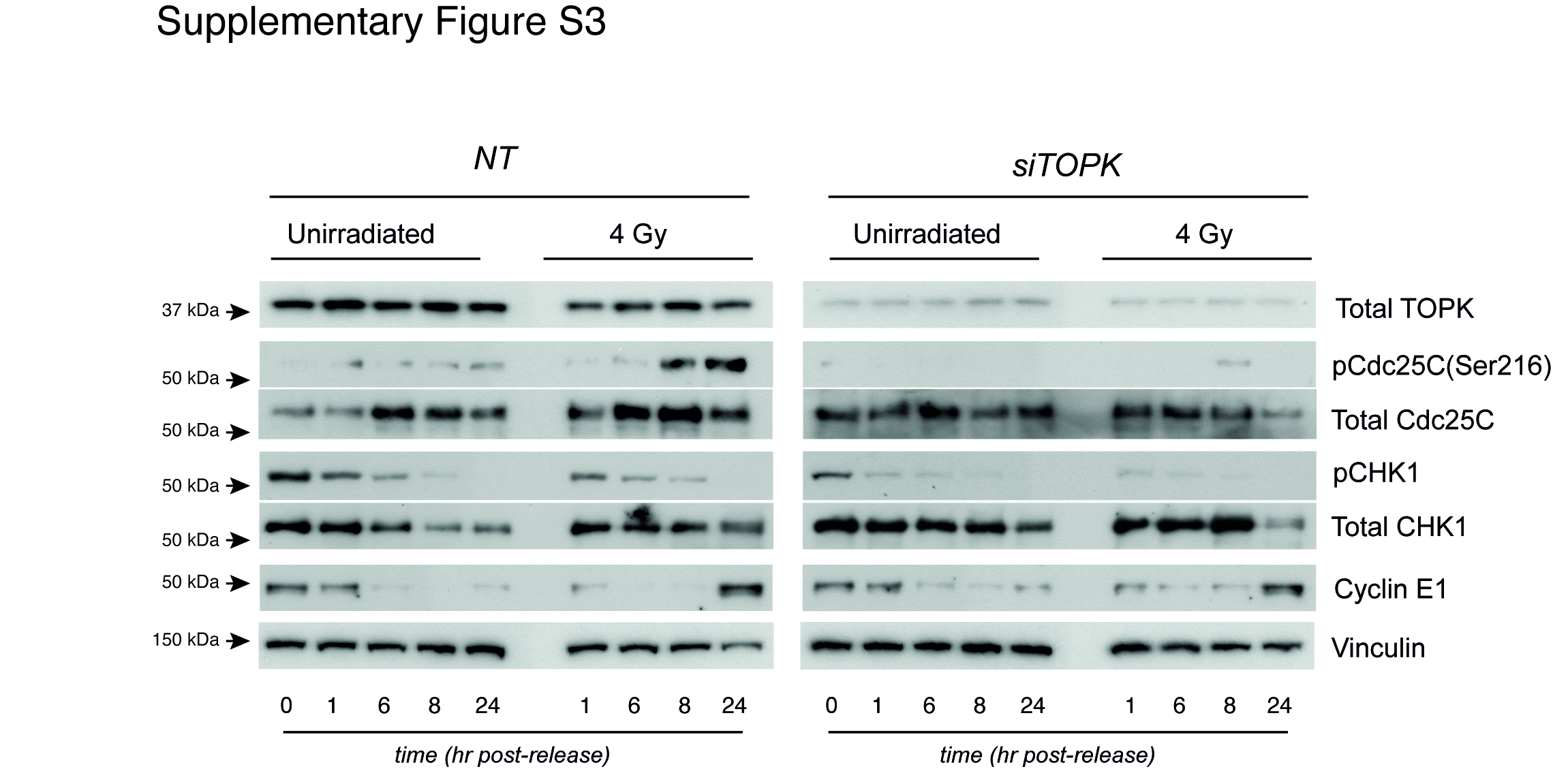

Supplement: Supplementary file 4 — Supplementary Figure S3 [file 41418_2020_655_MOESM4_ESM.tif]

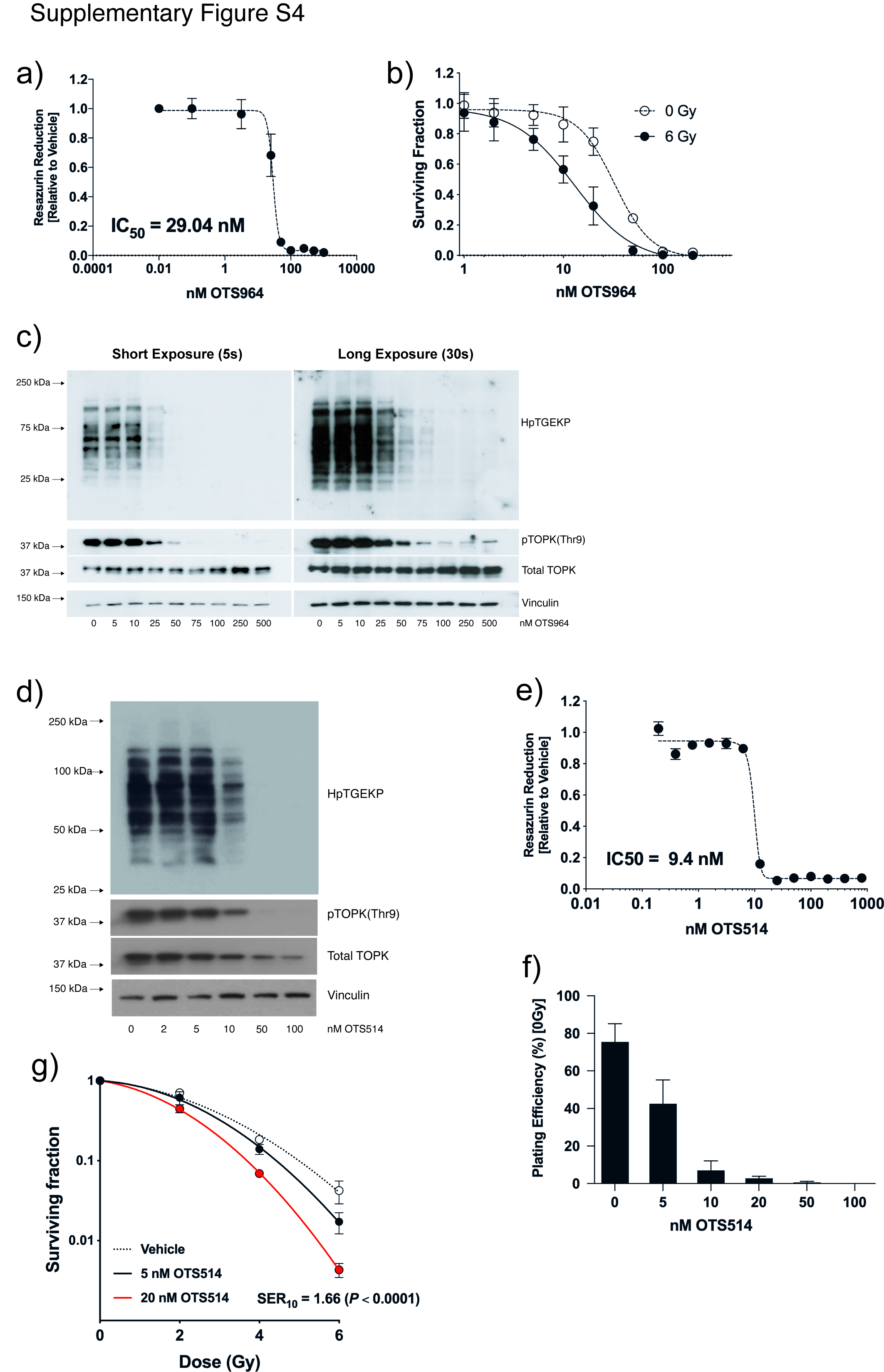

Supplement: Supplementary file 5 — Supplementary Figure S4 [file 41418_2020_655_MOESM5_ESM.tif]

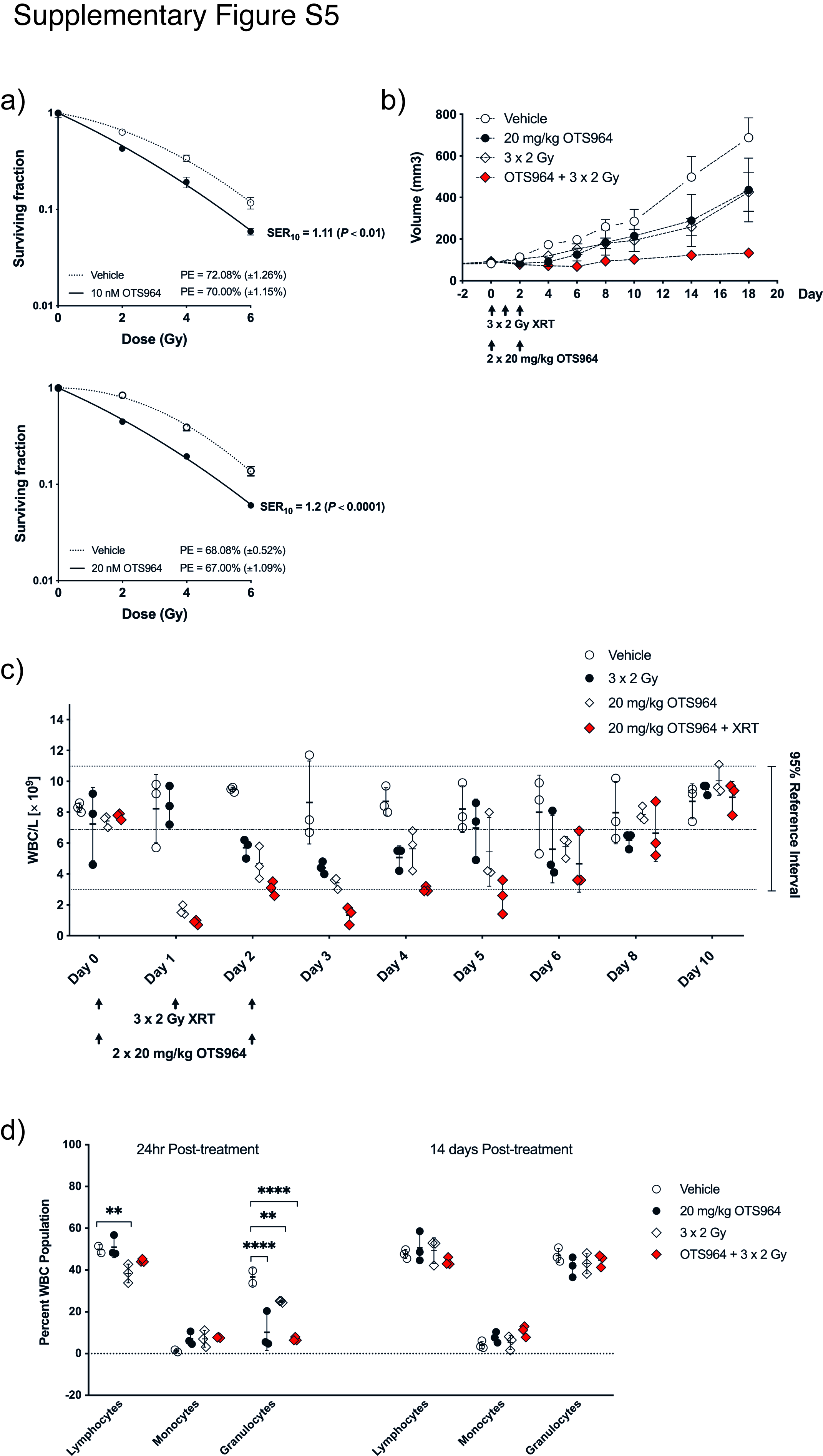

Supplement: Supplementary file 6 — Supplementary Figure S5 [file 41418_2020_655_MOESM6_ESM.tif]
